# Qualitative *De Novo* Analysis of Full Length cDNA and Quantitative Analysis of Gene Expression for Common Marmoset (*Callithrix jacchus*) Transcriptomes Using Parallel Long-Read Technology and Short-Read Sequencing

**DOI:** 10.1371/journal.pone.0100936

**Published:** 2014-06-30

**Authors:** Makiko Shimizu, Shunsuke Iwano, Yasuhiro Uno, Shotaro Uehara, Takashi Inoue, Norie Murayama, Jun Onodera, Erika Sasaki, Hiroshi Yamazaki

**Affiliations:** 1 Laboratory of Drug Metabolism and Pharmacokinetics, Showa Pharmaceutical University, Machida, Tokyo, Japan; 2 Novartis Pharma K.K., Tokyo, Japan; 3 Pharmacokinetics and Bioanalysis Center, Shin Nippon Biomedical Laboratories, Ltd., Kainan, Wakayama, Japan; 4 Department of Applied Developmental Biology, Central Institute for Experimental Animals, Kawasaki, Japan; 5 Eurofins Genomics, Ohta-ku, Tokyo, Japan; 6 Advanced Research Center, Keio University, Tokyo, Japan; The Perinatal Institute, Cincinnati Children's Hospital Medical Center and University of Cincinnati, United States of America

## Abstract

The common marmoset (*Callithrix jacchus*) is a non-human primate that could prove useful as human pharmacokinetic and biomedical research models. The cytochromes P450 (P450s) are a superfamily of enzymes that have critical roles in drug metabolism and disposition via monooxygenation of a broad range of xenobiotics; however, information on some marmoset P450s is currently limited. Therefore, identification and quantitative analysis of tissue-specific mRNA transcripts, including those of P450s and flavin-containing monooxygenases (FMO, another monooxygenase family), need to be carried out in detail before the marmoset can be used as an animal model in drug development. *De novo* assembly and expression analysis of marmoset transcripts were conducted with pooled liver, intestine, kidney, and brain samples from three male and three female marmosets. After unique sequences were automatically aligned by assembling software, the mean contig length was 718 bp (with a standard deviation of 457 bp) among a total of 47,883 transcripts. Approximately 30% of the total transcripts were matched to known marmoset sequences. Gene expression in 18 marmoset *P450*- and 4 *FMO*-like genes displayed some tissue-specific patterns. Of these, the three most highly expressed in marmoset liver were *P450 2D-*, *2E*-, and *3A*-like genes. In extrahepatic tissues, including brain, gene expressions of these monooxygenases were lower than those in liver, although *P450 3A4* (previously *P450 3A21*) in intestine and *P450 4A11*- and *FMO1*-like genes in kidney were relatively highly expressed. By means of massive parallel long-read sequencing and short-read technology applied to marmoset liver, intestine, kidney, and brain, the combined next-generation sequencing analyses reported here were able to identify novel marmoset drug-metabolizing P450 transcripts that have until now been little reported. These results provide a foundation for mechanistic studies and pave the way for the use of marmosets as model animals for drug development in the future.

## Introduction

The common marmoset (*Callithrix jacchus*) is a non-endangered member of the New World non-human primate family Callitrichidae native to northern and eastern Brazil [1]–[5]. It has attracted considerable attention as a potentially useful animal model in fields such as neuroscience, stem cell research, drug toxicology, immunity and autoimmune diseases, reproductive biology, and regenerative medicine [2] because of its size, availability, and unique biological characteristics [1]. One of the advantages of the common marmoset as an animal model for biomedical research is that its cells exhibit cross-reactivity with human cytokines and hormones [4]. The common marmoset has similar disease susceptibility and toxicological profiles to humans, making it a suitable model system for toxicology screening and drug development [1], [2], [6]. The teratogenic effects of some compounds have been shown to differ significantly between rodent and primate species. For example, whereas rodents are not sensitive to the teratogenic action of thalidomide analogues, the common marmoset is sensitive to these compounds [7]–[9].

Recently, the common marmoset has attracted much attention in biomedical research; however, genomic information for the common marmoset is not yet complete. Currently, the draft sequence assembled by the Washington University St. Louis (WUSTL) School of Medicine Genome Sequencing Center in St. Louis, Missouri, USA, and the Baylor College of Medicine (BCM) Human Genome Sequencing Center in Houston, Texas, USA, are available through NCBI GenBank (https://www.hgsc.bcm.edu/non-human-primates/marmoset-genome-project). The common marmoset genome was sequenced to 6× coverage using DNA from a female marmoset provided by the Southwestern National Primate Research Center in San Antonio, Texas, USA, assuming 95% coverage of the whole genome. To date, there are 292,992 expressed sequence tags (ESTs) available for the common marmoset in GenBank; however, this remains insufficient for the comprehensive understanding of common marmoset transcriptomes. Many low-expression transcripts would be missed from currently available EST data, which makes it difficult for analysis of marmoset transcriptomes to proceed further.

**Figure 1:**
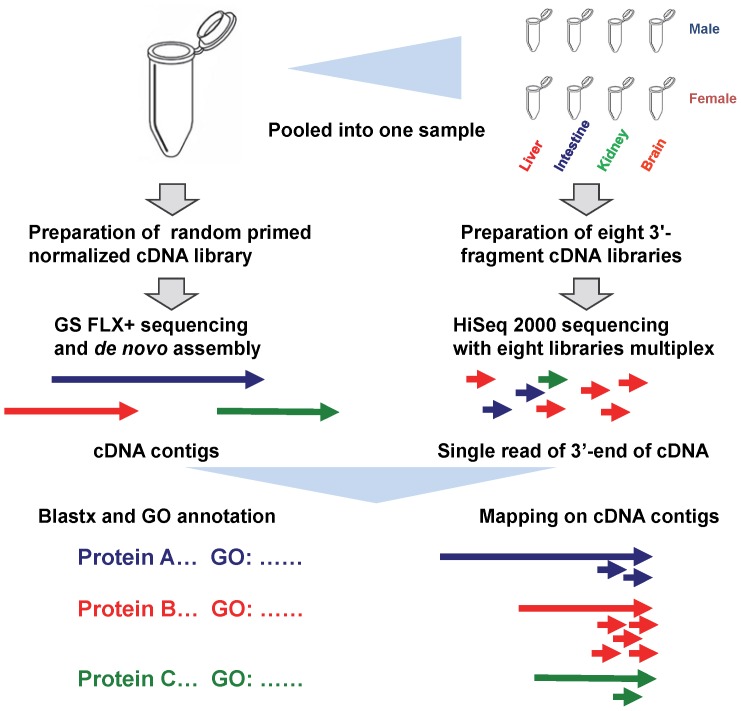
Dual transcriptomic strategy for qualitative *de novo* analysis of cDNA and quantitative analysis of gene expression. The procedures included sample preparation, cDNA library construction, sequencing, and data analysis that involved *de novo* assembly, application of BLAST software, gene ontology (GO) annotation, and elucidation of gene expression.

The fields of transcriptomics and genome characterization have developed rapidly with the advent of next-generation high-throughput sequencing technologies [such as the Illumina (Illumina), 454 (Roche) and SOLiD (ABI) platforms] in recent years [10]–[12]. Next-generation high-throughput RNA sequencing technology (RNA-seq) is a recently developed method for discovering, profiling, and quantifying RNA transcripts. RNA-seq has several advantages over other expression profiling technologies, including higher sensitivity and the ability to detect splicing isoforms and somatic mutations [13]. Because it is not restricted by the absence of an available reference genome sequence, this approach has been applied in decoding the genomes of several non-model organisms, providing valuable information on gene function, cell responses, and evolution [14]–[16]. The countable, almost digital, nature of RNA-seq data makes the technique particularly attractive for the quantitative analysis of transcript expression levels, yielding reliable measurements of transcript levels under one or more conditions [17]. The useful combination of massive parallel long-read sequencing technology and short-read technology [18] has been suggested for identification and quantitative analysis of mRNA transcripts and understanding gene expression, as summarized in Fig. 1. Such investigations in the common marmoset have yet to be reported.

Cytochrome P450 (P450, EC 1.14.14.1) enzymes have been characterized with respect to drug metabolism and disposition. Research has elucidated human liver microsomal P450 isoform contents [19]; their relatively broad but selective substrate specificities [20], [21]; and genetic polymorphisms, induction, and inhibition of human P450 enzymes [22]. The human *P450* gene superfamily is made up of 57 functional genes and 58 pseudogenes [23]. Research has focused on the P450s as well as on another monooxygenase family, flavin-containing monooxygenases [24] (FMO, EC 1.14.13.8), involved in the oxidation of a variety of compounds associated with pharmacological and/or toxicological actions. The knowledge gained so far in this field has been impressive, but many challenges remain.

By using massive parallel long-read sequencing and short-read technology with pooled liver, intestine, kidney, and brain samples from three male and three female common marmosets, the present study combined next generation sequencing analyses to identify novel common marmoset P450 transcripts that have until now been little reported. After unique sequences were automatically aligned by assembling software, the mean contig length was 718 bp (with a standard deviation of 457 bp) among a total of 47,883 transcripts. The gene expression of 18 common marmoset *P450*-like genes and 4 *FMO*-like genes that play critical roles in drug metabolism and disposition via monooxygenation of xenobiotics displayed some tissue-specific patterns. The results of this study will be an important resource for future biomedical research and will facilitate the use of the common marmoset as an animal model in new drug development.

## Materials and Methods

### Ethics Statement

This study was approved by the Institutional Animal Care and Use Committee of the Central Institute of Experimental Animals (No. 12025 and 13071) and Showa Pharmaceutical University (P-2012-01) and were performed in accordance with Guidelines for Proper Conduct of Animal Experiments by Science Council of Japan (2006). All animals were handled in strict accordance with good animal practice under the supervision of veterinarians. In accordance with the recommendations of the Weatherall report on the use of non-human primates in research, every effort was made to alleviate animal discomfort and pain by appropriate and routine use of anesthetic and/or analgesic agents.

### Animals

All tissue sample collections from three male and female common marmosets (CLEA Japan Inc., Tokyo Japan) after euthanasia by exsanguination under ketamine (60 mg/kg) and isoflurane deep anesthesia. Animal care was conducted in accordance with the recommendation of the Guide for the Care and Use of Laboratory Animals (Institute for Laboratory Animal Resources, 2011). The marmosets were housed in cages (409×610×1578 mm) in an environmentally controlled room under the temperature of 24–27°C and 40–60% relative air humidity with a 12/12 h light/dark cycle and had free access to a balanced diet (CMS-1M; CLEA Japan) added with vitamins and water. Wood perches for locomotion and gouging and a wooden platform for bed were placed in each cage for environmental enrichment.

### RNA Isolation, Library Preparation, and Sequencing

Total RNA was extracted from liver, intestine, kidney, and brain tissues of three male and three female common marmosets using an RNeasy Mini Kit (Qiagen, Valencia, CA, USA) according to the manufacturer's protocols. The concentrations and quality of total RNA samples were quantified by e-Spect (Malcom, Tokyo, Japan) and were evaluated for a 260/280 nm ratio >1.8, indicating high purity. Eight RNA samples of each tissue from male and female common marmosets were pooled to detect their gene expression profiles.

Library preparations and sequencing were performed at Eurofins Genomics (Ebersberg, Germany), where a dual transcriptomic strategy was employed that consisted of qualitative *de novo* analysis of cDNA and quantitative analysis of gene expression. This strategy is summarized in Fig. 1.

**Figure 2:**
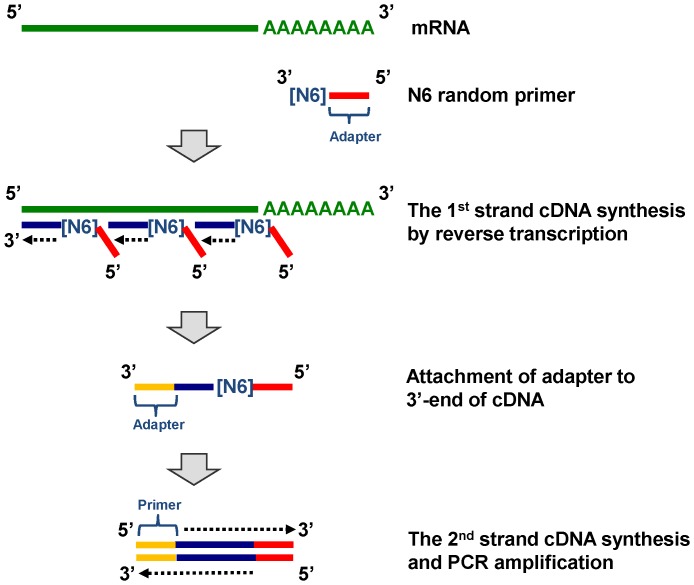
Synthesis of double-strand cDNA for sequencing. First strand cDNA synthesis was accomplished by randomized primers, which enabled mRNA to be covered from the poly(A) side to the 5′-side near the start point of transcription.

For cDNA sequencing, eight total RNA samples were pooled in equal quantities, and the preparation of a normalized cDNA library for Roche GS FLX+ sequencing was carried out as follows: poly(A)+ RNA was isolated from the above RNA pool, and first-strand cDNA synthesis was primed with a N6 randomized primer. Because they can anneal everywhere on mRNA stochastically, these randomized primers were expected to synthesize single-strand cDNA, which covered from the poly(A) to the 5′ side near the start point of transcription. Then 454 adapters were ligated to the 5′ and 3′ ends of the cDNA. Finally the cDNA was amplified using PCR and became double-strand cDNA (Fig 2). Normalization was carried out by one cycle of denaturation and re-association of the cDNA, and re-associated ds-cDNA was separated from the remaining ss-cDNA (normalized) by passing the mixture through a hydroxyl apatite column. The ss-cDNA was polymerase-chain-reaction (PCR) amplified, and the cDNA library in the size range of 500–1500 bp was eluted from a preparative agarose gel. Emulsion PCR and sequencing were conducted according to Roche standard protocols, and the normalized cDNA library was sequenced in 1/2-plate run of GS FLX+.

For quantitative analysis of gene expression, eight total RNA samples were prepared and 3′-fragment cDNA libraries were generated for each sample individually before application of Illumina HiSeq 2000 sequencing as follows. Each RNA sample was fragmented by sonication, and then poly(A)+ RNA was isolated from the fragmented total RNA. First-strand cDNA synthesis was performed using an oligo(dT)-adapter primer and reverse transcription. The resulting 3′-fragment cDNA was PCR amplified, and the cDNA library in the size range of 200–450 bp was eluted from a preparative agarose gel. The 3′-frgament cDNA library has 3′-SAGE like feature, therefore 3′-fragments can be regarded as 3′-tags without considering each cDNA length for the calculation of expression amounts. Cluster formation and sequencing were conducted according to Illumina standard protocols, and eight 3′-fragment cDNA libraries were sequenced in multiplex in one lane of a HiSeq 2000 with a 1×100-bp read module.

### Bioinformatics Analysis

*De novo* assembly of the single-read data of normalized cDNA sequencing was performed by MIRA Assembler Version 3.4. Contig sequences were annotated using Blast2GO software which takes a query collection of nucleotide sequences and uses the BlastX algorithm to search a UniProt database by gene ontology (GO). We used an expect value of 1E^−10^ for the BlastX searches. When the searches yielded multiple hits of annotations, the annotation with the best score was adopted for further analysis. WEGO was used to perform GO classifications and construct the GO tree [25]. All contig sequences in this study were also annotated using BlastN Version 2.2.29+ algorithm with human (taxid: 9606) database of NCBI Transcript Reference Sequences (refseq-rna) for cross validation.

Mapping of the single-read data of 3′-fragment cDNA sequencing was performed by BWA software using the above contig sequences as the reference. The numbers of mapped reads on contigs were considered to represent the levels of gene expression. To enable direct comparisons between the eight samples, the read numbers per reference were normalized based on the sample with the smallest number of total mapped reads of the eight samples analyzed.

## Results and Discussion

### Long Sequencing and Assembly

**Figure 3:**
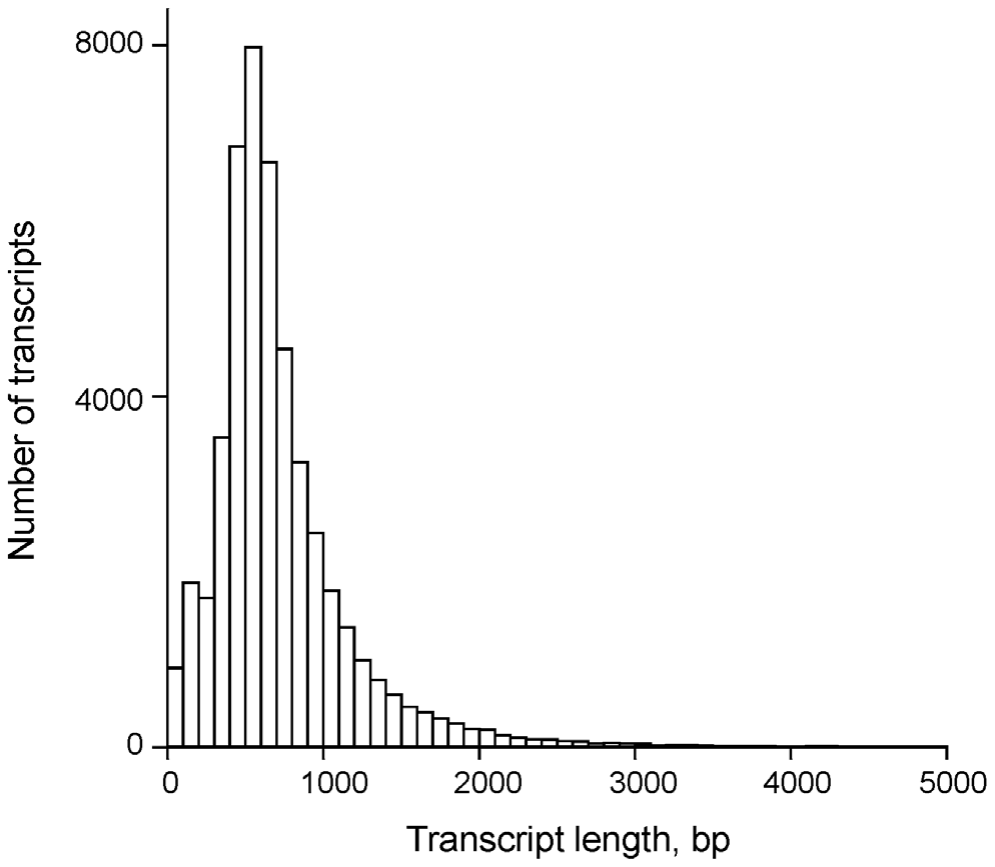
Assembly length statistics for marmoset sequences. Among the 47,883 transcripts, the mean length and SD values were 718 and 457: the longest gene was 7,339 bp in this study.

We performed Roche GS FLX+ sequencing of a normalized cDNA library prepared from four different tissues from three male and three female common marmosets to develop a comprehensive understanding of the molecular mechanisms governing common marmoset genome biology and to obtain as many gene transcripts as possible. Roche GS FLX+ sequencing generated 580,349 reads with an average length of 365 bp (212,277,507 bp of data in total); these were filtered at the standard of Q10 (Q10 is the quality score and means a sequencing error rate of <10%). The high-quality data were aligned and *de novo* assembled using MIRA Assembler Version 3.4 into 47,883 contigs consisting of 34,382,501 bp. Contigs ranged in size from 40 to 7,339 bp with an average length of 718 bp and an N50 length of 799 bp (Table 1). Among these contigs, 33,047 (69.0%) were longer than 500 bp, and 8,184 (17.1%) of this subset were longer than 1,000 bp, as shown in Fig. 3.

**Table 1:** Summary of sequence assembly.

Total entries	Total length, bp	Mean length, bp	N50, bp	Max length, bp	Min length, bp
47,883	34,382,501	718	799	7,339	40

GS FLX+ sequencing has been successfully used for *de novo* assembly of transcriptomes in many species [26]-[36]. In common with other recent studies, our results indicated that the GS FLX platform can provide much more data than the traditional Sanger sequencing method. The average size of the contigs in our study was 718 bp (Table 1), which was similar to those generated in previous studies using the GS FLX platform (e.g., 526 [26], 438 [27], 581 [28], 916 [29], 424 [30], 408 [31], 1,000 [34], and 583 [36]).

### Annotation of Contigs

Table 2 shows major species and the respective numbers of annotated contigs identified in this study. Based on the BlastX algorithm, 8,029 of the 47,883 contigs (17% of the total) had no annotation with known protein sequences. Under the present conditions, only 13,679 contigs (29% of the total) were annotated with common marmoset proteins. The second most common match was with human proteins [7,981 contigs (17%)]. A further 15% of contigs, i.e., 2,261 (5%), 1,680 (4%), 1,084 (2%), 865 (2%), 521 (1%), and 490 (1%), were annotated with known protein sequences from other primate family members. It should be noted that 959 contigs (2%) were annotated with mouse protein sequences. This poor overall annotation efficiency could have resulted from the relatively small number of sequences in public databases for the common marmoset. The numbers of non-annotated contigs of 8,029 (17%) were decreased to 3,449 (7.2%) after BlastN search with human trascriptomic database.

**Table 2:** Annotation statistics of contigs of common marmoset (*Callithrix jacchus*) transcripts.

Species	Numbers	% of total
*Callithrix jacchus*	13,679	29
*Homo sapiens*	7,981	17
*Macaca mulatta*	2,261	5
*Saimiri boliviensis*	1,680	4
*Macaca fascicularis*	1,084	2
*Mus musculus*	959	2
*Pan troglodytes*	865	2
*Pongo abelii*	521	1
*Nomascus leucogenys*	490	1
Others, annotated	10,334	22
Non-annotated	8,029	17

### Expression Analysis by Short Sequencing and GO Classification

**Figure 4:**
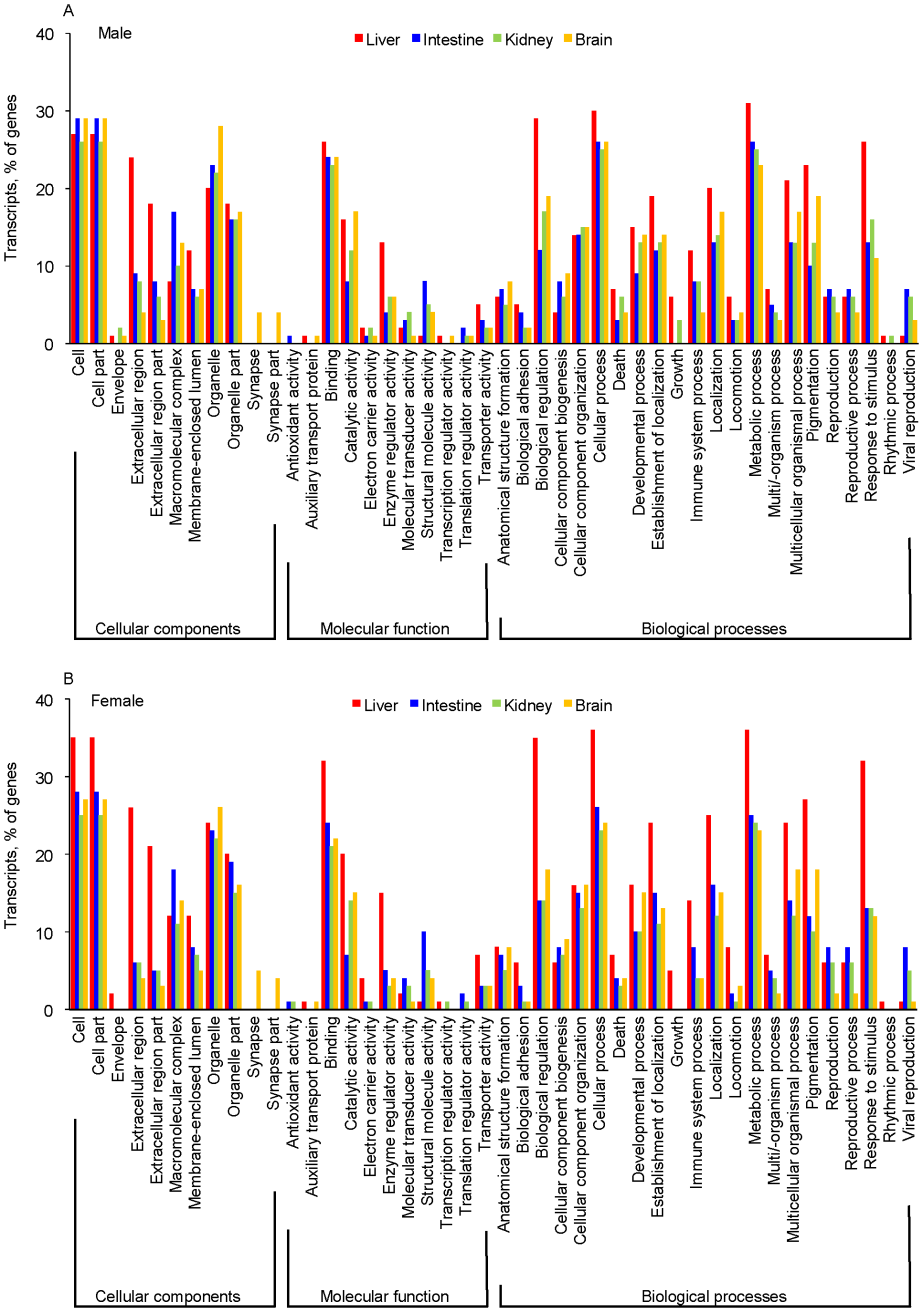
Gene Ontology (GO) classification of marmoset (*Callithrix jacchus*) sequences based on predicted gene ontology terms in liver, intestine, kidney, and brain from male (A) and female (B) marmosets. A total of 47,883 contigs with BLAST matches to known proteins were assigned to three main categories: cellular components, molecular function, and biological processes. RNA sequencing was done for mRNAs from liver, intestine, kidney, and brain of three male and three female marmosets. Only the top 100 genes are shown in this GO analysis.

For gene expression (amount) analysis, 3′-fragment library sequencing, focused on 200–450 bp range of 3′-end of transcripts, was adopted in this study. This unique 3′-UTR region sequencing strategy was expected to bring both benefits, rigorous identification of gene families and high sensitivity for expression counting. A total of 47,883 contigs with BLAST matches to known proteins were assigned to three main categories: cellular components, molecular function, and biological processes as shown in Fig. 4. mRNA expressions in liver, intestine, kidney, and brain of three male and three female common marmosets obtained by Illumina HiSeq 2000 sequencing were broadly similar when the top 100 genes were compared in GO analysis in this study; the top 10 transcripts identified or predicted by GO among the most abundantly expressed 100 genes in the tissues are shown in Table 3. The synapse and synapse part genes in the cellar component category and the response to stimulus genes in the biological process category were detected predominantly in the brain and liver, respectively (Fig. 4), suggesting that GO analysis might reflect the appropriate function of each tissue.

**Table 3:** Top 10 transcripts identified or predicted by GO annotation among the most abundant 100 contigs in tissues from male and female common marmosets.

	Liver	Intestine	Kidney	Brain
Male	1. C3 and PZP-like alpha-2-macroglobulin domain-containing protein 1 [*Macaca mulatta*]	1. tetraspanin-8*	1. cytochrome *c* oxidase subunit III	1. hsp90aa1 protein [*Homo sapiens*]
	2. cytochrome *c* oxidase subunit III	2. cytochrome *c* oxidase subunit III	2. immunoglobulin heavy chain [*Homo sapiens*]	2. histone demethylase uty-like [*Homo sapiens*]
	3. angiotensinogen precursor	3. apolipoprotein A-IV*	3. translationally-controlled tumor protein-like [*Saimiri boliviensis boliviensis*]*	3. histone demethylase UTY-like [*Pan troglodytes*]*
	4. protein AMBP*	4. achain model of human iga1 [*Homo sapiens*]	4. glutathione peroxidase 3*	4. cytochrome *c* oxidase subunit III
	5. alpha-1-antitrypsin precursor-like protein	5. 60S ribosomal protein L13a-like [*Pan troglodytes*]*	5. 60S ribosomal protein L13a-like [*Pan troglodytes*]*	5. E3 ubiquitin-protein ligase TTC3*
	6. inter-alpha-trypsin inhibitor heavy chain H4 isoform 1 [*Gorilla gorilla gorilla*]*	6. apolipoprotein A-I*	6. phosphoenolpyruvate carboxykinase, cytosolic [GTP]*	6. thy-1 membrane glycoprotein*
	7. translationally-controlled tumor protein-like [*Saimiri boliviensis boliviensis*]*	7. translationally-controlled tumor protein-like [*Saimiri boliviensis boliviensis*]*	7. NADH dehydrogenase subunit 1	7. NADH dehydrogenase subunit 1
	8. inter-alpha-trypsin inhibitor heavy chain H3 [*Saimiri boliviensis boliviensis*]*	8. immunoglobulin heavy chain [*Homo sapiens*]	8. heat shock protein HSP 90-beta [*Equus caballus*]	8. heat shock protein HSP 90-beta [*Equus caballus*]
	9. hemopexin*	9. 60S acidic ribosomal protein P0-like isoform 2*	9. insulin-like growth factor-binding protein 1*	9. glyceraldehyde-3-phosphate dehydrogenase*
	10. zinc-alpha-2-glycoprotein-like*	10. fructose-bisphosphate aldolase B*	10. glyceraldehyde-3-phosphate dehydrogenase*	10. cytoplasmic dynein 1 heavy chain 1*
Female	1. C3 and PZP-like alpha-2-macroglobulin domain-containing protein 1 [*Macaca mulatta*]	1. achain model of human iga1 [*Homo sapiens*]	1. immunoglobulin heavy chain [*Homo sapiens*]	1. cytochrome *c* oxidase subunit III
	2. cytochrome *c* oxidase subunit III	2. cytochrome *c* oxidase subunit III	2. cytochrome *c* oxidase subunit III	2. hsp90aa1 protein [*Homo sapiens*]
	3. protein AMBP*	3. tetraspanin-8*	3. translationally-controlled tumor protein-like [*Saimiri boliviensis boliviensis*]*	3. NADH dehydrogenase subunit 1
	4. inter-alpha-trypsin inhibitor heavy chain H4 isoform 1 [*Gorilla gorilla gorilla*]*	4. immunoglobulin heavy chain [*Homo sapiens*]	4. glutathione peroxidase 3*	4. heat shock protein HSP 90-beta [*Equus caballus*]
	5. alpha-1-antitrypsin precursor-like protein	5. 60S ribosomal protein L13a-like [*Pan troglodytes*]*	5. phosphoenolpyruvate carboxykinase, cytosolic [GTP]*	5. histone demethylase UTY-like [*Pan troglodytes*]*
	6. translationally-controlled tumor protein-like [*Saimiri boliviensis boliviensis*]*	6. apolipoprotein A-IV*	6. 60S ribosomal protein L13a-like [*Pan troglodytes*]*	6. E3 ubiquitin-protein ligase TTC3*
	7. beta-2-glycoprotein 1-like isoform 1*	7. translationally-controlled tumor protein-like [*Saimiri boliviensis boliviensis*]*	7. glyceraldehyde-3-phosphate dehydrogenase*	7. thy-1 membrane glycoprotein*
	8. immunoglobulin heavy chain [*Homo sapiens*]	8. hsp90aa1 protein [*Homo sapiens*]	8. protein NDRG1 isoform 1*	8. Putative calmodulin, partial [*Desmodus rotundus*]
	9. betaine-homocysteine methyltransferase, isoform CRA_b [*Homo sapiens*]	9. MHC class I Caja-G*05, partial	9. prostaglandin F synthase 1-like*	9. glyceraldehyde-3-phosphate dehydrogenase*
	10. aldehyde dehydrogenase, mitochondrial isoform 1 [*Saimiri boliviensis boliviensis*]*	10. apolipoprotein A-I*	10. fructose-bisphosphate aldolase B*	10. synaptotagmin-1 isoform 7 [*Macaca mulatta*]*

*Predicted.

### Expression of Monooxygenases in Liver, Intestine, Kidney and Brain of the Common Marmoset

**Figure 5:**
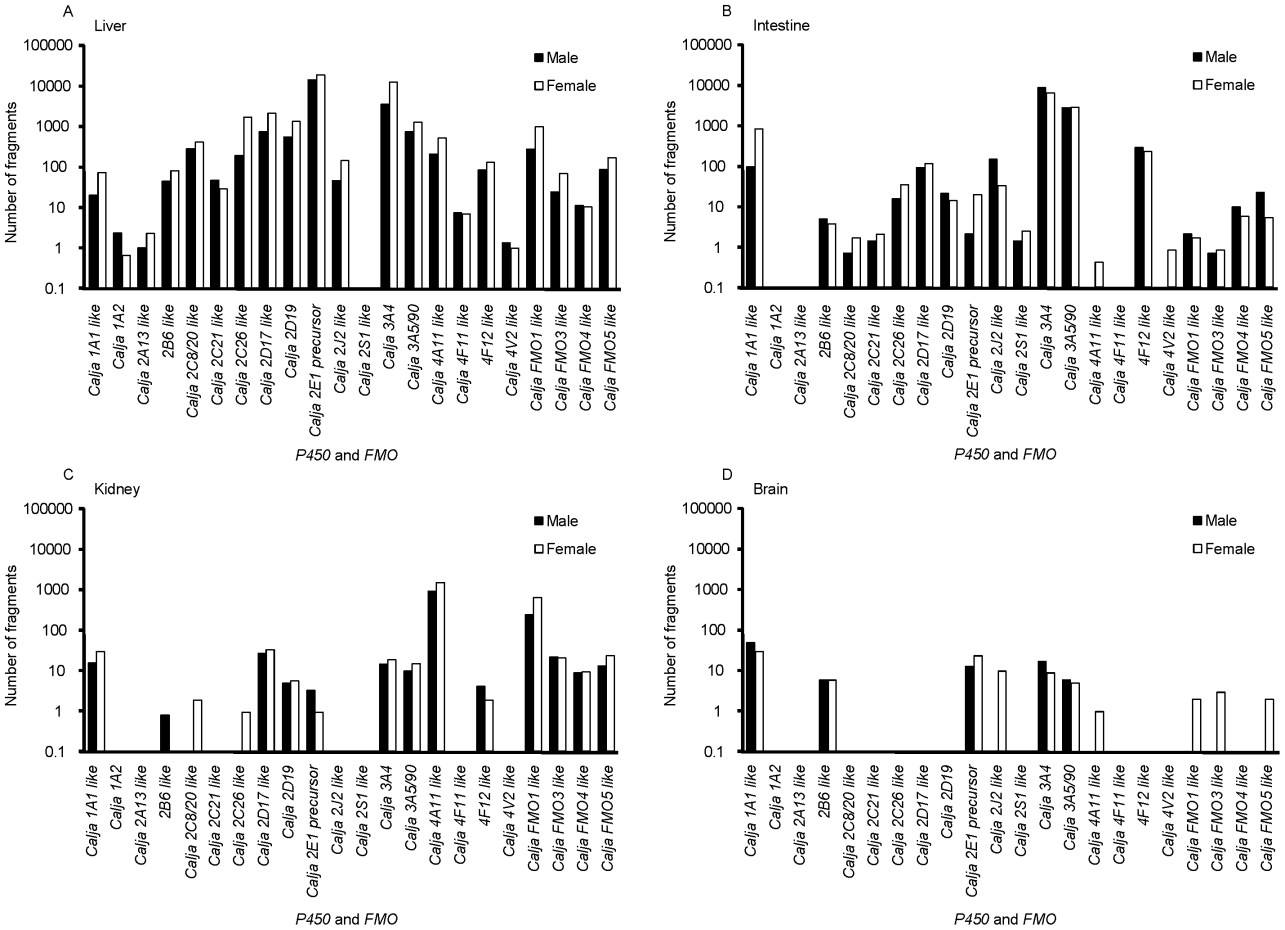
Expression profiles of marmoset *P450* and *FMO* genes in liver (A), intestine (B), kidney (C), and brain (D) from male and female marmosets. RNA sequencing was done for mRNAs from liver, intestine, kidney, and brain of three male (black bars) and three female (open bars) marmosets. The mRNA abundance was expressed as the number of fragments. Bars represent the means of pooled tissue samples from three individual marmosets. Known marmoset (*Callithrix jacchus*) P450 enzymes are shown as Calja P450. The others are named as *P450*- or *FMO*-like based on gene annotation.

Some drug-metabolizing enzymes were detected in the present GO analysis. Drug-metabolizing P450 and FMO enzymes are essential for activation and deactivation of drugs, medicines, and environmental toxicants. In this study, 18 forms of P450 and 4 forms of FMO were detected (Fig. 5A-5D). It should be noted that P450 3A5 and 3A90 were annotated as 3A5/90 because of the substantially high sequence homology of their cDNAs (approximately 97%). Among the currently known common marmoset P450s, the gene expression of marmoset *P450 1A2, 2B6, 2C8, 2D19, 2E1*, *3A4* (previously named *P450 3A21*), and *3A5/90* enzymes was detected in liver (Fig. 5A). Other sequences highly homologous to human *P450* or *FMO* were annotated as *P450* or *FMO* “like.” In male and female livers, *Callithrix jacchus* (Calja) P450 2E1 transcripts were the most abundant of all P450s, and Calja P450 3A4 was the second most abundant, followed by Calja P450 2D17-like, 2C26-like, 2D19, 3A5/90, and Calja FMO1-like transcripts (Fig. 5A). Recently, cDNA resources for the common marmoset have been developed [37]. The present contigs from marmoset P450 1A1, 1A2, 2A13-like, 2B6-like, 2C8/20-like (these have 97% homology), 2C21-like, 2C26-like, 2D17-like, 2D19, 2E1, 3A4, 3A5/90 and FMO1 and FMO3 were hit with the reported ESTs, suggesting that the reported major marmoset P450 and FMO enzymes were covered in this study.

In extrahepatic tissues (Fig. 5B–5D), monooxygenase gene expression was generally less abundant than in liver, the major drug-metabolizing organ. In intestine, Calja P450 3A4 was highly expressed, followed by Calja P450 3A5/90 (Fig. 5B). In kidney, Calja *P450 4A11*-like and Calja *FMO1*-like genes were highly expressed (Fig. 5C). Although gene expression levels of P450s and FMOs in the brain were generally lower than in other tissues, Calja *P450 1A1*-like gene was more abundantly expressed than other P450s and FMOs (Fig. 5D).

Calja *P450 3A4* gene was abundantly expressed in liver (Fig. 5A). Calja *P450 3A4* is orthologous to human and cynomolgus *P450 3A4* [38], based on its genomic location in the *P450 3A* gene cluster [39]. This gene was previously named *P450 3A21*, but is referred to as *P450 3A4* in this article. *P450 3A4* gene is also abundantly expressed in cynomolgus macaque livers [40]. P450 3A4 is one of the most important drug-metabolizing enzymes because it is involved in the oxidation of more than half of all prescription drugs. Moreover, P450 3A4 is the most abundant P450 in human liver [19] and cynomolgus macaque liver [41]. The abundant expression of *P450 3A4* gene in common marmoset liver and intestine raises the possibility that P450 3A4 might be one of the most abundant P450s in common marmoset liver, just as it is in human and cynomolgus macaque liver.

Calja *P450 3A4* gene was also abundantly expressed in intestine (Fig. 5B), just as cynomolgus P450 3A4 gene is in cynomolgus intestine [40]. P450 3A4 is the most abundantly expressed P450 in human small intestine [42]. The abundant expression of P450 3A4 in liver and intestine might indicate similarities in the first-pass effect of drug metabolism among marmosets, cynomolgus macaques, and humans. In common marmoset liver and intestine, *P450 3A5/90* gene was also abundantly expressed (Fig. 5B). Because P450 3A4 shares some substrates with P450 3A5 in humans [43] and in cynomolgus macaques [44], it is possible that marmoset P450 3A5/90 might also metabolize some P450 3A4 substrates, contributing to overall drug metabolism in liver and intestine. It would be of great interest to investigate the protein expressions and metabolic properties of P450 3A4 and 3A5/90 in common marmoset liver and intestine; the information obtained would help elucidate the disposition of new drugs in common marmosets.

### Confirmation of Representative Tissue-specific mRNA Expressions in the Common Marmoset

Tissue-specific mRNA expressions were confirmed in this study by investigating selected known drug transporters. Table 4 shows expression profiles of some transporters in liver, intestine, kidney, and brain in common marmosets. Solute carrier organic anion transporter family member (SLCO) 1B3-like protein and *Calja* SLCO1B3 were, respectively, predominantly expressed and mostly expressed in common marmoset livers, similar to the suggested profiles in humans [45]. Solute carrier family (SLC) 5A1 and ATP-binding cassette (ABC) sub-family G member 2-like protein were abundant in common marmoset intestines, just as they are in human intestines [46]. SLC22 expression levels in common marmoset kidney were higher than those for other SLC family members, as has also been reported in humans [47]. SLC1A3-like protein and another drug-metabolizing enzyme sulfotransferase (SULT) 4A1 were, respectively, predominantly and abundantly expressed in marmoset brains, similar to the case in human brains [48], [49]. These results suggested that representative tissue-specific RNA expressions of transporter and drug metabolizing proteins were reflected in the present combined next-generation sequencing analyses.

**Table 4:** Representative expression profiles of tissue-specific transporter genes in liver, intestine, kidney, and brain from male and female marmosets.

Transporter/enzyme	Number of fragments							
	Liver		Intestine		Kidney		Brain	
	M	F	M	F	M	F	M	F
SLCO1B1-like	27	40	0	0	0	0	0	4
*Calja* SLCO1B3	344	643	0	0	63	94	3	0
*Calja* SLC5A1	0	1	2051	1688	120	59	0	1
*Calja* ABCG2-like	8	9	56	40	3	11	2	7
*Calja* SLC22A2	0	0	0	0	2254	3468	60	35
SLC1A3-like	0	0	0	0	0	0	3	18
*Calja* SULT4A1	0	0	6	2	1	0	699	1268

RNA sequencing was done for mRNAs from liver, intestine, kidney, and brain of three male (M) and three female (F) marmosets. The mRNA abundance was expressed as the number of fragments. Known marmoset (*Callithrix jacchus*) transporters are shown as *Calja* SLC. The others are named as *ABC*- or *SLC*-like based on gene annotation. SLCO, solute carrier organic anion transporter; SLC, solute carrier family; ABC, ATP-binding cassette; and SULT, sulfotransferase.

### Comparison of Common Marmoset Drug-metabolizing Enzymes with Those of Other Primates

In common marmoset liver, the metabolic activity of five P450 enzymes (P450 1A2, 2B6, 2C8, 2D19, and 2D30) have been characterized using recombinant proteins [50]–[52]. Marmoset P450 1A2, 2B6, and 2D19/30 metabolize the typical substrates of the orthologous human P450 isoforms, i.e., phenacetin, bupropion, and bufuralol, respectively [50]–[52], indicating some similarities in the metabolic properties of P450s between marmosets and humans. Paclitaxel is a typical substrate of human P450 2C8 that is also metabolized by cynomolgus P450 2C8 [53]; however, marmoset P450 2C8 has been reported not to metabolize paclitaxel [54]. Cynomolgus P450 1A1/2, 2B6, and 2D17/44 metabolize the human P450 substrates described above, indicating that the metabolic properties of P450s are similar in marmosets, cynomolgus macaques, and humans, but the cynomolgus macaque appears to be more similar to humans in terms of the metabolic properties of P450 2C8 than the marmoset is. It would be of great interest to investigate the remaining 12 P450s covered in the present study in terms of substrate specificities and metabolic capacities.

*P450 1A1*-like gene was more abundantly expressed than *P450 1A2* in marmoset liver, just as it was in intestine, kidney, and brain (Fig. 5). Similarly, cynomolgus *P450 1A1* gene is the most abundantly expressed *P450 1A* gene in liver and extra-hepatic tissues [40]. However, in humans, P450 1A2 is the major P450 1A in liver, whereas P450 1A1 is the major P450 1A in extra-hepatic tissues [55]. Thus, the tissue expression pattern of marmoset *P450 1A* genes appears to be more similar to cynomolgus *P450 1A* than to human *P450 1A*.

In common marmoset liver, *P450 2D17*-like and *P450 2D19* expression was found in the current study (Fig. 5A). A previous study reported the expression and function of another P450, 2D30, in common marmoset liver; however, the P450 2D30 cDNA was isolated from samples of one animal group, but not from another [50], indicating the possibility of differences in *P450 2D30* expression among animals or groups. This might be the reason why P450 2D30 was not detected in our study.

In common marmoset kidney, *P450 4A11*-like gene was the most abundantly expressed of all P450s (Fig. 5C), as is also the case for *P450 4A11* gene in cynomolgus kidney [56]. P450 4A11 is also expressed in human kidney [57]. *FMO1*-like gene was the most abundantly expressed of the FMOs in common marmoset kidney (Fig. 5C), and the same is true for the cynomolgus *FMO1* gene [58]. FMO1 is also the major FMO in human kidney [59]. In common marmoset intestine, *P450 2J2*-like and *4F12*-like genes were moderately expressed (Fig. 5B), just as they are in the cynomolgus macaque [56]. Expression of *P450 2J2* and *4F12* has also been found in human intestine [60].

Among the four *FMO* genes expressed in common marmoset liver, *FMO1*-like gene was the most abundantly expressed (Fig. 5A). Cynomolgus FMO1 is not substantially expressed in postnatal liver [58]; the same is true for human FMO1, which is expressed in fetal liver, but the expression disappears after birth [59]. In contrast, FMO1 is postnatally expressed in the liver of other species. FMO3 is the major FMO in liver in humans [59], just as it is in the cynomolgus macaque [58]. As a result of these species differences in FMO1 expression in liver, extrapolation of data obtained in these species to humans could be complicated. The cynomolgus macaque might be a better animal model than the common marmoset for the study FMO-dependent drug metabolism.

Sex differences in gene expression in common marmoset liver were noted for several P450s and FMOs. In common marmoset liver, expression of *P450 1A1*-like, *2C26*-like, *2D17*-like, *2J2*-like, *3A4*, and *3A5/90* genes and *FMO1*-like gene was higher (>2.5-fold) in females than in males, whereas *P450 1A2* gene was more abundantly expressed in males than in females (Fig. 5A). The sex difference in gene expression in common marmoset liver was most marked for *P450 3A4* gene, which was approximately 3.6-fold higher in females than in males. Similarly, hepatic expression of human P450 3A4 is higher in females than in males [61]. In rodents, sex-dependent expression of P450 genes is partly mediated by differences in the secretion pattern of pituitary growth hormone (GH) between males and females [62]. Moreover, sex hormones, including estradiol, have been shown to influence expression of *P450* genes [62], possibly resulting in sex-dependent expression of these genes. Therefore, GH and sex hormones play roles in sex differences in *P450* gene expression, and this may also be true for P450 3A4 in common marmoset liver.

In this transcriptomic study, we used total RNAs from only four organs: liver, kidney, intestine, and brain. Genes that are expressed in other specific organs or under different stimuli/conditions do not fall within the scope of this study. Therefore, the total numbers of contigs obtained in this qualitative transcriptome was judged to be a reasonable value. In future studies involving both determination of the whole common marmoset genome by *de novo* genome sequencing and identification of gene (exon) sequences using mRNA-sequencing and *ab initio* calculation, we expect that around 20,000 genes will be identified, as is the case in other mammalians. In conclusion, the present combination of parallel long-read sequencing technology and short-read technology revealed the mRNA abundance of all the P450s and FMOs in common marmoset liver, intestine, kidney, and brain. The combined next-generation sequencing analysis by means of massive parallel long-read sequencing and short-read technology reported here is considered to constitute one of novel methods for identifying new gene family transcripts for genes that have been little reported. The present results provide a foundation for the possible use of marmosets as model animals for mechanistic studies and drug development for humans in the future.

## Database Accession Number

The full length cDNA data by GS FLX+ sequencing reported in this paper will appear in the DDBJ Sequence Read Archive (DRA) under the accession number DRA002207.

## Supporting Information

Checklist S1 ARRIVE Guidelines Checklist.
(PDF)
